# Analysis of Adverse Events in the Treatment of Patients with Non-Valvular Atrial Fibrillation with Oral Anticoagulants: Data from the “ANTEY” Observational Study

**DOI:** 10.3390/ph15101209

**Published:** 2022-09-29

**Authors:** Sergey Yu. Martsevich, Yulia V. Lukina, Natalia P. Kutishenko, Anton R. Kiselev, Oxana M. Drapkina

**Affiliations:** 1 Department of Preventive Pharmacotherapy, National Medical Research Center for Therapy and Preventive Medicine, 101990 Moscow, Russia; 2 Coordinating Center for Fundamental Research, National Medical Research Center for Therapy and Preventive Medicine, 101990 Moscow, Russia; 3 National Medical Research Center for Therapy and Preventive Medicine, 101990 Moscow, Russia

**Keywords:** oral anticoagulants, atrial fibrillation, adverse events

## Abstract

Rationale. Therapy with oral anticoagulants (OACs) in patients with atrial fibrillation (AF) is based on finding the optimal balance of efficacy and safety of these drugs. Data from observational studies are an additional source of information for the adverse events (AEs) of pharmacotherapy. Objective: To investigate pharmacotherapy AEs with OACs in the “ANTEY” prospective observational study in patients with non-valvular atrial fibrillation (AF). Material and Methods: A total of 201 people were enrolled (83 (41.3%) were women). The age of subjects was 71.1 ± 8.7 years (data presented as mean with standard deviation). The study protocol included two face-to-face visits (contacts V0 and V1) and one follow-up (FU) phone contact which were made with the patient at an interval of 6 months. At V0, all patients were recommended to take one of the non-vitamin K antagonist oral anticoagulants (NOACs); starting from V1, warfarin could have been prescribed or NOAC could have been changed. Information about AEs and OACsadministration was collected at V0, V1, and FU. Results. During 1 year of observation, 15 out of 201 patients refused to take OACs, and 186 initiated the recommended drug. Rivaroxaban was initiated in 93 patients, dabigatran in 46, apixaban in 40, and warfarin in 7 patients. There were 55 AEs, 25 of which were serious (SAEs), including 4 deaths. Of the 30 AEs, there were 18 bleedings: eight (8.6%) occurred with the administration of rivaroxaban; four (8.5%) with dabigatran, three (7.5%) with apixaban, and three (42.9%) with warfarin. Differences in the incidence of bleeding events between NOACs and warfarin are statistically significant (*p* = 0.025). Any AEs increased the chance of nonadherence to treatment nine-fold: OR = 9.2 (CI95%: 3.6–23.5), *p* < 0.0001. Conclusions. The most typical and common AEs in real-world clinical practice settings treatment with OACs were bleedings, the incidence of which was approximately 8% to 9% in the treatment with NOACs and was much higher with warfarin, bleedings in the treatment with OACs are statistically significantly associated with nonadherence to the use of these drugs in the future.

## 1. Introduction

Pharmacotherapy is currently the most common medical intervention in the world. In chronic non-infectious diseases, pharmacotherapy, if supported by data from randomized controlled trials (RCTs) and evidence-based medicine, is the main method of improving the prognosis of the disease and prolonging the life of patients [1]. A major concern related to pharmacotherapy is its safety issues. Data from the American (Food and Drug Administration, FDA, USA) and European (European Medicines Agency, EMA, EU) regulatory agencies for the registration and safety surveillance of medicinal products indicate a significant number of adverse events (AEs) related to pharmacotherapy [2,3]. A significant number of AEs are identified during the post-registration stage and in observational studies. This can be explained by the longer duration of patient follow-up, the minimal limitations of concomitant therapy in real-world clinical practice settings, and the wider prescription of drugs to the categories of patients whose proportion in the RCTs was limited by the inclusion criteria.

Therapy with oral anticoagulants (OACs) is based on finding the optimal balance of efficacy and a reduction in the incidence of thromboembolic complications and safety in relation to bleeding as the most common side effects of these drugs. Currently, the issues of the efficacy and safety of drug therapy are often studied in conjunction with the aspect of adherence to treatment [4,5]. Thus, Yao et al. showed that a poor adherence to OACs increases the risk of stroke in patients with atrial fibrillation (AF), but reduces the likelihood of bleeding, the frequency of which is increased in adherent patients [6].

A prospective observational study, “ANTEY” (**A**ssessment of Adherence to **N**ew Oral an**T**icoagulants in Atrial Fibrillation pati**E**nts Within the Outpatient registr**Y**), investigated the adherence of patients with a non-valvular form of AF to non-vitamin K antagonist oral anticoagulants (NOACs) [7,8]. In this study, an additional analysis was carried out, the purpose of which was to study all AEs and unfavorable outcomes reported with OACs therapy to assess their relationship with OACs administration in patients with a non-valvular form of atrial fibrillation during one year of observation.

## 2. Results

During one year of observation, four deaths were reported; follow-up (FU) contact occurred in 197 patients. At V1, 160 (80.0%) patients took the NOACs recommended to them during the previous visit, 33 (16.5%) patients immediately refused to take NOACs, and 7 patients began but then discontinued NOACs for various reasons. At the FU contact, 158 (80.2%) of 197 patients were adherent to the recommended therapy. The remaining 39 people did not take OACs: 15 people refused to take it (primary non-adherence), and 24 people discontinued OACs (secondary non-adherence) [8]. These patients were considered non-adherent to treatment. Thus, during the observation period, 15 (7.5%) patients did not take OACs at all, and the remaining 186 (92.5%) patients either continued or discontinued taking OACs: dabigatran—46 patients, rivaroxaban—93 patients, apixaban—40 patients, and warfarin after V1—7 patients.

During the entire observation period, 55 cases of various AEs of pharmacotherapy were reported, 25 of which were serious adverse events (SAEs), including 4 deaths—sudden cardiac death, death from sarcoma, death from chronic heart failure, and death from an accident—and 30 AEs. All AEs, both serious and non-serious, and their relationship to OACs administration are presented in Table 1. Deaths are presented separate from other SAEs in this table, as listed in the ClinicalTrials.gov website.

**Figure 1:**
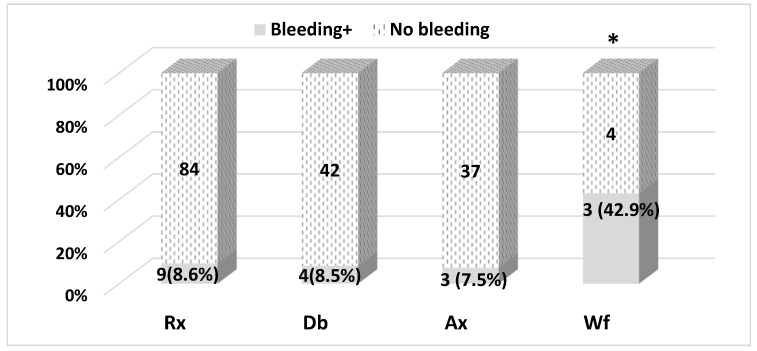
Incidence of bleedings with OACs therapy. Rx, rivaroxaban; Db, dabigatran; Ax, apixaban; Wf, warfarin. * *p* = 0.025 (warfarin vs. NOACs).

A total of 18 bleeding events were reported (Figure 1). Eight (8.6%) of the bleeding events occurred with Rivaroxaban (93 patients initiated the drug), four (8.6%) with dabigatran (46 patients), three (7.5%) with Apixaban therapy (40 patients), and three (42.9%) with Warfarin therapy (7 patients). There were differences between the number of bleeding events.

Differences in the number of bleeding events between NOACs are statistically non-significant (*p* = 0.920), and the difference between NOACs and warfarin is statistically significant: OR = 7.6 (CI95%: 1.6–37.2), *p* = 0.025 (two-tailed Fisher’s exact probability test). However, it should be noted that a small number of study patients took warfarin; therefore, caution is necessary in interpreting the clinical significance of the results obtained.

There were no severe bleeding events reported in the study, and all bleeding events were mild to moderate in severity according to the GUSTO Bleeding Classification [9].

**Figure 2:**
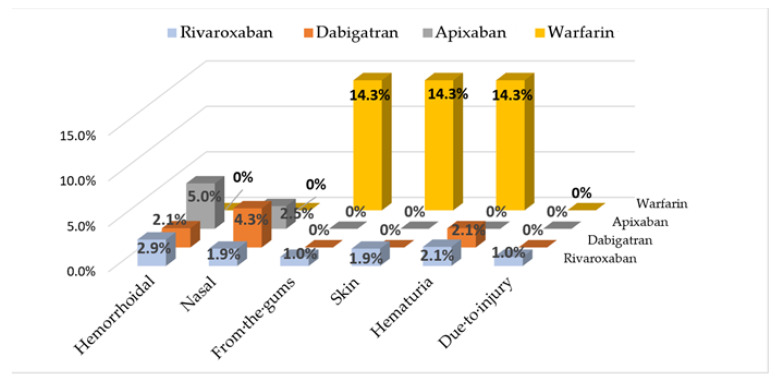
Incidence of bleeding sites with OACs therapy (% of patients who initiated one of the OACs).

Bleeding site location information for patients who initiated one of the OACs is presented in Figure 2. According to two-tailed Fisher’s exact probability test, the difference in the number of bleeding events between NOACs and warfarin is statistically significant: OR = 7.6 (CI95%: 1.6–37.2), *p* = 0.025.

Patients’ characteristics with bleeding and without bleeding developed during OACs therapy are presented in Table 2. Of note, among patients with developed bleeding, most had a high risk of bleeding according to the HAS-BLED scale: OR = 4.2 (CI95%: 1.4–12.3), *p* = 0.006 (two-tailed Fisher’s exact probability test); almost 90% of them had CHF.

The components of the HAS-BLED scale were distributed as follows: the greatest contribution to the increased risk of bleeding was made by age: OR = 5.9 (CI95%: 0.8–45.4), *p* = 0.08 (two-sided Fisher’s exact criterion); the presence of bleeding: OR = 6.0 (CI95%: 1.8–19.9), *p* = 0.007; stroke history: OR = 1.8 (CI95%: 0.6–5.9), *p* = 0.48; and alcohol abuse: OR = 2.6 (CI95%: 0.3–24.9), *p* = 0.38. The differences between the groups of patients with developed bleeding and without this complication are presented in Table 3.

Any pharmacotherapy AEs increased the chance of nonadherence to treatment nine-fold: OR = 9.2 (CI95%: 3.6–23.5), *p* < 0.0001. In the group of patients with bleedings reported with OACs administration, 83.3% of patients refused to take these drugs and only 16.7% continued the previously recommended therapy (*p* = 0.033). During the entire observation period, 80% of patients with non-valvular AF clearly followed the recommendations of the scientific center doctors regarding OACs administration [8,10].

## 3. Discussion

The issue of safety in pharmacotherapy is of great relevance in modern medicine and society. Its effective solution is complicated by the extreme difficulty of collecting complete information on pharmacotherapy AEs (adverse events). The leading sources of such information are RCT data, the results of observational studies, spontaneous reports submitted by physicians to pharmacovigilance authorities [11]. RCTs provide the most reliable data on safety of, as a rule, new medicinal products (MPs). Unfortunately, due to a number of features, they are unable to provide complete information on the safety of the MPs being studied. This is due to the relatively small number of patients enrolled in RCTs, the frequent exclusion of some patient groups (children, pregnant, elderly people, patients with severe chronic diseases, pronounced comorbidity, etc.), and the relatively short duration of follow-up of patients in RCTs compared to the intended active treatment period. This may result in very rare AEs or SAEs being undetectable in RCTs, despite the better reporting of AEs in RCTs compared to routine clinical practice. Therefore, approximately half of the MPs require a change to the official product label in the post-marketing stage.

Due to the identified safety concerns, approximately one in five MPs receives a “black box” in the official label warning about possible serious (sometimes life-threatening) AEs identified only in the post-marketing stage. It is known that about 4% of MPs are withdrawn from circulation for safety reasons [12,13].

The peculiarities of the way observational studies are conducted provide them with some advantages over RCTs in detecting AEs. These features of observational studies include: (1) real-world clinical practice settings for comparison groups; (2) a rather large population allowing the investigation of rare AEs; (3) a long follow-up duration; (4) the inclusion of different groups of patients, including those who are usually excluded from RCTs; and (5) evaluation of the results of actual use of MPs in both approved indications and “off-label” prescriptions.

The FDA began establishing a national real-world clinical practice data collection system from leading US medical centers in 2008, called the Sentinel Initiative, which became fully effective in 2016. The documentation of this project emphasizes that the conduct of post-marketing studies to investigate the safety of new MPs is as important as the conduct of clinical studies before the drugs enter the pharmaceutical market. During the period of 2008–2012, 385 post-marketing studies were initiated by the FDA, resulting in changes to the labels of 65 medicinal products [14].

The “ANTEY” study is classified as “initiated by investigators”. The AEs data obtained in it confirmed the high safety of NOACs, which caused significantly less bleedings compared to warfarin, as well as a higher adherence of patients with AF to taking these drugs [7,8]. This is consistent with both the results of both RCTs with NOACs [15,16,17] and the data from observational studies [18,19,20].

However, according to the results of the Ko YJ study, based on the Korea Institute of Drug Safety and Risk Management–Korea database, it was found that rivaroxaban was associated with the more frequent development of bleeding events than treatment with dabigatran or apixaban [21]. The results of our study and data from a number of other studies did not identify any significant differences between the different NOACs in the incidence of bleeding [22,23].

The results of the study confirmed the good predictive ability of the HAS-BLED scale to predict the occurrence of bleeding in patients with AF in real clinical practice: in the presence of a high risk for HAS-BLED, the chance of bleeding increased 4-fold. The results of the study demonstrated that bleeding was more common in patients with CHF. Perhaps the reason for this was the older age of these patients. In addition, it is well-known that patients with heart failure have a high prevalence of renal dysfunction [22]. Since all NOACs are renally eliminated to various degrees while warfarin is not, progressive renal function decline leads to an increased risk of bleeding in the treatment of NOAC [24,25]. However, in our study there was only one patient with renal dysfunction (according to the criteria of the HAS-BLED scale).

It should be emphasized that the results of the “ANTEY” study show a high persistence (patient adherence to long-term treatment) of patients to the administration of NOACs: 80% of patients with non-valvular AF took the recommended OACs during the 1 year of observation. The high incidence of bleedings with warfarin may be explained by failures to adhere to the rules for its administration.

The reduction in bleeding risk with NOACs treatment could be achieved by (1) adherence to clinical guidelines; (2) a risk assessment of the HAS-BLED scale and exposure to modifiable risk factors (hypertension, alcohol abuse); and (3) for avoiding the co-administration with drugs that increase the risk of bleeding, for example, NSAIDs.

According to the Institute of Safe Medicine Practices, OACs has been identified as high-risk drugs. Bleeding is the most typical AE of OACs; it is directly related to their actions and occurs in 8–19% of treated patients per year [26,27]. In a Norwegian study of 65,000 patients taking NOACs and 80,000 patients taking warfarin, the most common AEs were hemorrhages (48% for direct-acting oral anticoagulants and 75% for warfarin) [28]. The results of our study are consistent with these data: twenty AEs, for which the relationship to OACs administration was determined, included 18 bleedings.

## 4. Material and Methods

The detailed design and protocol of the “ANTEY” study are described in earlier publications [7,8]. The study is registered at www.clinicaltrials.gov (accessed on 26 September 2022) (ANTEY Trial Identifier: NCT03790917). The study was conducted in accordance with the guidelines of the Declaration of Helsinki and approved by the Institutional Ethics Committee of National Medical Research Center for Therapy and Preventive Medicine (protocol No. 05-01/17, 3 August 2017). All patients signed an informed consent form for the processing of personal data and participation in the “ANTEY” study.

The “ANTEY” study is part of the PROFILE Outpatient Prospective Registry, which was established on the basis of a specialized cardiology department. The “ANTEY” study enrolled all patients with non-valvular AF on this registry. Exclusion criteria were absolute contraindications to OACs, according to the official product labels for NOACs and warfarin. Thus, 201 people were enrolled, 83 (41.3%) of whom were women, with a mean age of 71.1 ± 8.7 years (data presented as mean with standard deviation) [7].

There were three groups of patients included in the study: (1) patients who had never taken NOACs before (n = 55); (2) patients who had previously taken warfarin (n = 21); and (3) patients who had taken one of the NOACs (n = 125).

The study protocol included two face-to-face visits (contacts V0 and V1) and one follow-up (FU) phone contact, which were made with the patient at an interval of 6 months. At V0, all patients were recommended to take one of the non-vitamin K antagonist oral anticoagulants (NOACs): dabigatran, rivaroxaban, or apixaban. For patients of the third group (patients who took one of the NOACs before V0), the attending physician could recommend the same drug, changing its dosage and frequency of administration if necessary, or could replace it with another NOAC.

Starting from V1, warfarin could be prescribed or NOAC could be changed. Information about AEs and OACs administration was collected at V0, V1, and FU.

Information about OACs adherence and data on all AEs were obtained from the patients during contacts V0 and V1 and FU. An AE was defined as any unfavorable event happening to a patient during participation in this study. The relationship between drug intake and AEs was established by a physician according to a known scale: definite, probable, possible, unlikely, no relation. Bleeding severity was assessed using the GUSTO Bleeding Classification scale. Bleedings leading to hemodynamic impairment and requiring intervention were considered severe [9].

Statistical processing was performed using the SPSS Statistics software package (IBM) version 23.0 (IBM, Armonk, NY, USA). Absolute values and percentages were used for descriptive statistics. The comparison of AEs with different OACs (NOACs and warfarin) was performed using Pearson’s chi-squared test with Yates’s correction for continuity and two-tailed Fisher’s exact probability test. The odds ratio (OR) and 95% confidence interval (CI95%) were calculated using Mantel–Haenszel statistics for dichotomous variables. Differences were considered statistically significant at *p* < 0.05.

## 5. Conclusions

The results of prospective observational studies provide more comprehensive information on the safety of MPs in the post-registration stage and identify AEs not reported in RCTs.

The “ANTEY” observational study showed that the most typical and common AEs in the real-world clinical practice settings treatment with OACs were bleedings, the incidence of which was approximately 8% to 9% in the treatment with NOACs. Bleedings during OACs therapy are statistically significantly associated with nonadherence to the use of these drugs in the future.

## 6. Strengths and Limitations

The advantage of the “ANTEY” observational study is its prospective nature, the performance of the study based on a scientific medical center with highly qualified medical personnel prescribing therapy according to clinical guidelines.

The limitations of the study are that it was a single-center study with a small number of enrolled patients; therefore, this study was likely underpowered for detecting major bleeding with a frequency of approximately 1–2%.

## Figures and Tables

**Table 1:** Adverse events and relationship to OACs administration.

Adverse Events	Number of Cases	AEs Relationship to OACs Administration
Deaths	4	
Cardiovascular death	1	Unrelated
Death from CHF	1	Unrelated
Death from sarcoma	1	Unrelated
Death from an accident	1	Unrelated
SAEs	21	
AMI	1	Unrelated
Unstable angina	5	Unrelated
Decompensation of CHF	5	Unrelated
AF paroxysm	5	Unrelated
Cerebral stroke	2	Unrelated
Retinal hemorrhage	1	Unrelated *
Severe iron deficiency anemia	1	Unlikely
Sarcoma	1	Unrelated
AEs	30	
Bleeding	18	Definite
Nausea	4	Unrelated
Urticaria	2	Unrelated
Constipation	2	Probable
Hypothyroidism	1	Unrelated
Cough	1	Unrelated
Gynecomastia	1	Unrelated
Hypotension	1	Unrelated

CHF, chronic heart failure; AMI, acute myocardial infarction; AF, atrial fibrillation; AEs, adverse events; SAEs, serious adverse events; OACs, oral anticoagulants. * Patient did not take OAC.

**Table 2:** Comparative characteristics of patients with and without bleeding.

Patients’ Characteristics		Bleeding (n = 18)	No Bleeding (n = 183)	Statistical Significance
HAS-BLED score	<3 (low risk)	5 (27.8%)	113 (61.7%)	*p* = 0.005
	≥0 (high risk)	13 (72.2%)	70 (38.3%)	
CAD	no	9 (50.0%)	88 (48.1%)	*p* = 0.877
	yes	9 (50.0%)	95 (51.9%)	
CHF	no	2 (11.1%)	100 (54.6%)	*p* < 0.0001
	yes	16 (88.9%)	83 (45.4%)	
DM	no	12 (66.7%)	129 (70.5%)	*p* = 0.735
	yes	6 (33.3%)	54 (29.5%)	

HAS-BLED, hypertension, abnormal liver/renal function, stroke history, bleeding history or predisposition, labile international normalized ratio, elderly, drug/alcohol usage; CAD, coronary artery disease; CHF, chronic heart failure; DM, diabetes mellitus.

**Table 3:** Clinical characteristics of the HAS-BLED score in patients with and without bleeding.

HAS-BLED Factors		Bleeding (n = 18)	No Bleeding (n = 183)	Statistical Significance
Hypertension Uncontrolled > 160 mmHg systolic	no	2 (11.1%)	11 (6.0%)	*p* = 0.27
	yes	16 (88.9%)	172 (94.0%)	
Renal disease Dialysis, transplant, Cr > 2.26 mg/dL or >200 µmol/L	no	18 (100.0%)	182 (99.5%)	*p* = 0.753
	yes	0	1 (0.5%)	
Liver disease Cirrhosis or bilirubin > 2x normal with AST/ALT/AP > 3x normal	no	18 (100.0%)	183(100.0%)	*p* = 1.0
	yes	0	0	
DM	no	12 (66.7%)	129 (70.5%)	*p* = 0.735
	yes	6 (33.3%)	54 (29.5%)	
Stroke history	no	14	158 (86.3%)	*p* = 0.15
	yes	4	25 (13.7%)	
Prior major bleeding or predisposition to bleeding	no	13 (72.2%)	172 (94.0%)	*p* = 0.029
	yes	5 (27.8%)	11 (6.0%)	
Labile INR Unstable/high INRs, time in therapeutic range < 60%	no	18 (100%)	181 (98.9%)	*p* = 0.656
	yes	0	2 (1.1%)	
Age > 65	no	1 (5.6%)	47 (25.7%)	*p* = 0.056
	yes	17 (94.4%)	136 (74.3%)	
Medication usage predisposing to bleeding Aspirin, clopidogrel, NSAIDs	no	11 (61.1%)	115 (62.8%)	*p* = 0.885
	yes	7 (38.9%)	68 (37.2%)	
Alcohol use ≥8 drinks/week	no	17 (94.4%)	179 (97.8%)	*p* = 0.381
	yes	1 (5.6%)	4 (2.2%)	

HAS-BLED, hypertension, abnormal liver/renal function, stroke history, bleeding history or predisposition, labile INR, elderly, drug/alcohol usage; Cr, creatinine; AST, aspartate aminotransferase; ALT, alanine transaminase; AP, alkaline phosphatase; DM, diabetes mellitus; INR, international normalized ratio; NSAIDs, non-steroidal anti-inflammatory drugs.

## Data Availability

Not applicable.
